# Genome-Wide Identification and Bioinformatics Analysis of the *FAD* Gene Family in Walnut (*Juglans regia* L.)

**DOI:** 10.3390/ijms27177879

**Published:** 2026-09-03

**Authors:** Fan Hao, Jingchuan Xia, Zhenlin Shen, Shuoxin Zhang

**Affiliations:** 1 College of Forestry, Northwest A&F University, Yangling 712100, China; 2 Qinling National Forest Ecosystem Research Station, Huoditang, Ankang 711600, China; 3 Key Laboratory of Resource Biology and Biotechnology in Western China, Ministry of Education, College of Life Sciences, Northwest University, Xi’an 710069, China

**Keywords:** walnut, fatty acid desaturase, gene family, bioinformatics

## Abstract

Fatty acid desaturase (*FAD*) is a core catalytic enzyme in plants for the synthesis of unsaturated fatty acids, profoundly affecting plant growth, development, and adaptability to various environmental stresses. The walnut (*Juglans regia* L.) is an important woody oil tree species, and its kernel is rich in unsaturated fatty acids. Systematic identification of the walnut *FAD* gene family and analysis of its function are of great significance for revealing the molecular mechanisms underlying unsaturated fatty acid metabolism in the walnut. Based on walnut whole-genome data, this study used homology alignment and hidden Markov model search methods to identify the *JrFAD* gene family members. Subsequently, a variety of bioinformatics tools were used to systematically analyze their structural characteristics, evolutionary expansion mechanism, expression regulation, and function. A total of 21 *JrFAD* gene family members were identified and classified into five subfamilies. The family genes were unevenly distributed on nine chromosomes. WGD/segmental duplication was the main expansion method, and the duplicated gene pairs experienced strong purification selection. The family gene promoter sequence is rich in regulatory elements that respond to light, plant hormones, and various stresses. The expression pattern analysis showed that *JrFAD3.1* and *JrFAD2.3* showed high expression specifically during the rapid accumulation of walnut kernel oil. This study clarified the composition and evolutionary characteristics of the *FAD* gene family in the walnut, which provides useful information for in-depth analyses of its functional mechanism in the regulation of lipid metabolism, and also identified potential candidate gene resources for the genetic improvement of walnut varieties with high amounts of unsaturated fatty acids.

## 1. Introduction

Fatty acids and their derived lipids are important energy storage molecules and structural components of biological membranes in plant cells, playing key roles in plant growth and development, signal transduction, and stress responses [1,2]. According to the presence and number of double bonds in their carbon chains, fatty acids can be classified as saturated fatty acids, monounsaturated fatty acids, or polyunsaturated fatty acids [3]. The content and composition of unsaturated fatty acids directly affect their membrane lipid structure, membrane fluidity, and membrane stability, further participating in plant adaptation to environmental changes such as low temperature, high temperature, drought, salt stress, and pathogen infection [4,5,6,7].

Fatty acid desaturases (*FADs*) are key enzymes that regulate the degree of fatty acid unsaturation. They catalyze the formation of C=C double bonds at specific positions of fatty acyl chains, thereby promoting the biosynthesis of monounsaturated and polyunsaturated fatty acids [4]. Based on protein solubility and subcellular localization, plant *FADs* are mainly divided into soluble *FADs* and membrane-bound *FADs* [8]. Among them, soluble *FADs* are mainly represented by stearoyl–ACP desaturases (*SAD/FAB2*), which are localized in the plastid stroma and convert stearic acid (C18:0) into oleic acid (C18:1) [9]. Membrane-bound *FADs* are more complex and generally include several subfamilies, such as *FAD4*, *FAD2/FAD6*, *FAD3/FAD7/FAD8*, and *ADS/SLD/DES* [10]. *FAD2* and *FAD6* belong to the ω-6 fatty acid desaturases and mainly catalyze the conversion of oleic acid to linoleic acid (C18:2), whereas *FAD3*, *FAD7*, and *FAD8* belong to the ω-3 fatty acid desaturases and further catalyze the conversion of linoleic acid to α-linolenic acid (C18:3) [11]. In terms of subcellular localization, *FAD2* and *FAD3* are mainly localized in the endoplasmic reticulum, whereas *FAD4*, *FAD6*, *FAD7*, and *FAD8* are mostly localized in plastids or chloroplast membrane systems [7,12,13].

In recent years, *FAD* gene families have been systematically identified in various plant species. These studies have shown that *FAD* genes are not only involved in fatty acid biosynthesis and seed oil accumulation but also closely associated with plant stress responses [5,10,11]. For example, wheat (*Triticum aestivum* L.) *TaFAD8* affects chloroplast membrane stability and heat stress adaptation by regulating the C18:2/C18:3 ratio and lipid remodeling; natural variation in its promoter can alter the basal expression level of *TaFAD8-D* and improve wheat thermotolerance [6]. In addition, potato (*Solanum tuberosum*) *StFAD7* responds significantly to low-temperature stress and may participate in cold-induced lipid remodeling and oxidative stress alleviation [7]. Therefore, elucidating the structural characteristics, evolutionary relationships, and expression patterns of the *FAD* gene family will help improve our understanding of fatty acid metabolism regulation and oil quality formation in plants.

The walnut (*Juglans regia* L.) is a traditional woody oil tree species widely cultivated worldwide, possessing high economic and nutritional value [14,15]. Its kernels contain 60–70% fat, and its oil consists of approximately 90% unsaturated fatty acids, including essential fatty acids such as linoleic acid and α-linolenic acid [16,17]. These fatty acids play important roles in promoting human growth and development, regulating blood lipid metabolism, and preventing cardiovascular diseases [18,19]. With the continuous improvement of walnut genome resources, it remains necessary to systematically identify and analyze the *JrFAD* gene family at the genome-wide level. In particular, the chromosomal distribution, gene structure, conserved motifs, phylogenetic relationships, duplication and expansion patterns, promoter regulatory elements, and expression characteristics across different tissues and kernel developmental stages require further clarification. In this study, genome-wide identification of *JrFAD* gene family members was performed based on walnut genome data. Their physicochemical properties, phylogenetic relationships, gene structures, chromosomal distribution, collinearity relationships, selection pressure, promoter *cis*-acting elements, and expression patterns were systematically analyzed. The results provide a basis for elucidating the molecular mechanisms underlying unsaturated fatty acid biosynthesis and oil quality formation in the walnut, and offer valuable references for potential candidate gene mining and the genetic improvement of walnut cultivars with high oil contents and high unsaturated fatty acid levels.

## 2. Results

### 2.1. Identification of the JrFAD Gene Family and Analysis of Protein Physicochemical Properties

A total of 21 *FAD* gene family members were identified in the walnut genome through homology-based BLAST searches, HMM domain searches, and conserved domain validation. According to their chromosomal locations and phylogenetic relationships, these genes were designated as *JrFAB2.1–JrFAB2.5*, *JrFAD2.1–JrFAD2.3*, *JrFAD3.1–JrFAD3.3*, *JrFAD4*, *JrFAD6*, *JrFAD7*, *JrFAD8*, *JrADS*, *JrSLD1–JrSLD3*, and *JrDES1–JrDES2*. The protein sequences of all identified *JrFAD* members are listed in Table S1. The predicted JrFAD proteins ranged from 307 to 455 amino acids in length, with *JrFAD4* being the shortest and *JrFAD8* the longest. Their molecular weights ranged from 34.68 to 51.77 kDa. The theoretical isoelectric points varied from 6.10 to 9.95, and 18 members had pI values greater than 7, indicating that most JrFAD proteins are alkaline. The instability indices ranged from 30.09 to 54.50, with 14 members predicted to be stable proteins based on instability index values below 40. The aliphatic index ranged from 74.49 to 95.69, while the grand average of hydropathicity values ranged from −0.480 to 0.061. Among them, 18 JrFAD proteins had GRAVY values below 0, suggesting that most members are hydrophilic proteins (Table S2).

Subcellular localization prediction revealed distinct predicted localization patterns among the *JrFAD* members. Members of the *FAB2* subfamily were mainly predicted to be localized in chloroplasts, whereas *FAD2* and *FAD3* subfamily members were mainly predicted to be localized in the endoplasmic reticulum. Members of the *SLD*, *DES*, and *ADS* subfamilies were primarily predicted to be localized in the plasma membrane. These results indicate that JrFAD proteins may differ in their physicochemical properties and subcellular localization. However, CELLO predictions are not always consistent with known biology. For instance, CELLO assigned the plastid-stromal *JrFAB2.1* to mitochondria, and predicted ER-localized *SLD/DES* members to the plasma membrane. These discrepancies suggest systematic biases of CELLO for certain *FAD* classes. Therefore, all predictions require caution and experimental validation. The secondary structures of the 21 JrFAD proteins were further predicted using SOPMA. All JrFAD proteins were composed of four structural elements: α-helices, β-turns, extended strands, and random coils (Figure S1). Among these elements, α-helices accounted for 34.37–63.24% and random coils accounted for 31.31–56.26%, representing the predominant structural components of JrFAD proteins. In contrast, β-turns and extended strands accounted for relatively small proportions, ranging from 0.94% to 3.89% and from 2.06% to 13.17%, respectively. These results suggest that JrFAD proteins are mainly characterized by α-helices and random coils.

### 2.2. Phylogenetic Relationships of the JrFAD Gene Family

**Figure 1:**
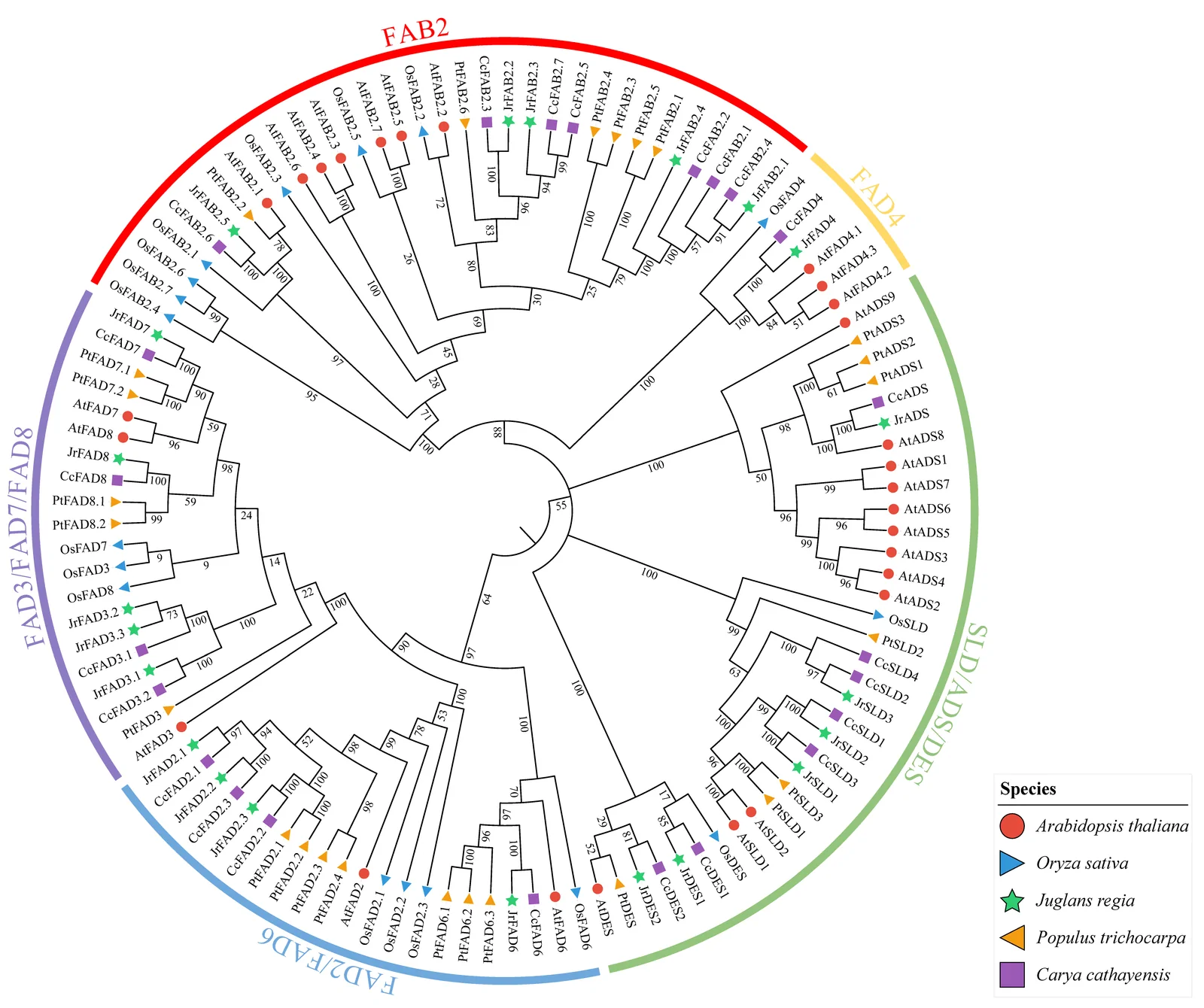
Phylogenetic tree of *FAD* gene family members from *Juglans regia*, *Arabidopsis thaliana*, *Oryza sativa*, *Populus trichocarpa*, and *Carya cathayensis*. Sequences from *Juglans regia*, *Arabidopsis thaliana*, *Oryza sativa*, *Populus trichocarpa*, and *Carya cathayensis* are represented by green five-pointed stars, red circles, blue triangles, orange triangles, and purple squares, respectively. The phylogenetic tree resolves FAD proteins into five distinct clades, *FAB2*, *FAD4*, *ADS/SLD/DES*, *FAD2/FAD6*, *and FAD3/FAD7/FAD8*, with each clade distinguished by a different color.

To clarify the evolutionary relationships of the *JrFAD* gene family, FAD protein sequences from walnut, Arabidopsis (*Arabidopsis thaliana*), rice (*Oryza sativa*), black cottonwood (*Populus trichocarpa*), and hickory (*Carya cathayensis*) were used to construct a phylogenetic tree. The protein sequences used for the phylogenetic analysis are listed in Table S3. The *JrFAD* members were classified into five major subfamilies: *FAB2*, *FAD4*, *ADS/SLD/DES*, *FAD2/FAD6* (ω-6), and *FAD3/FAD7/FAD8* (ω-3) (Figure 1). This classification was generally consistent with the *FAD* gene family classification reported in model plants, indicating that the *FAD* gene family is highly conserved during plant evolution. The *FAB2* subfamily contained 32 members, comprising five from walnut, seven from Arabidopsis, seven from rice, six from black cottonwood, and seven from hickory. Members of this subfamily are mainly associated with the conversion of stearic acid (18:0) to oleic acid (18:1) [20]. The *FAD4* subfamily contained six members, comprising one from walnut, three from Arabidopsis, one from rice, and one from hickory, while no *FAD4* subfamily members were found in black cottonwood. This subfamily and is mainly involved in the desaturation of palmitic acid (16:0) to palmitoleic acid (16:1) in plastid membranes [21]. The *ADS/SLD/DES* subfamily contained 34 members, comprising six from walnut, 12 from Arabidopsis, two from rice, seven from black cottonwood, and seven from hickory, and may primarily participate in the metabolism of fatty acid derivatives and sphingolipids [11,22]. The *FAD2/FAD6* (ω-6) subfamily contained 21 members, comprising four from walnut, two from Arabidopsis, four from rice, seven from black cottonwood, and four from hickory. These proteins mainly catalyze the introduction of a double bond at the Δ-12 position of oleic acid (C18:1), generating linoleic acid (C18:2) [23]. The *FAD3/FAD7/FAD8* (ω-3) subfamily contained 20 members, comprising five from walnut, three from Arabidopsis, three from rice, five from black cottonwood, and four from hickory, and mainly participates in the conversion of linoleic acid (C18:2) to α-linolenic acid (C18:3) [24].

Beyond this overall classification, further examination of the topological details revealed that within each subfamily, walnut FAD proteins generally clustered more closely with their orthologs from black cottonwood and hickory than with those from Arabidopsis or rice, reflecting the closer phylogenetic relationships among these woody dicot species. For instance, in the *FAD2/FAD6* subfamily, *JrFAD2.1*–*JrFAD2.3* grouped with *CcFAD2.1*–*CcFAD2.3* and *PtFAD2.1*–*PtFAD2.4* within a well-supported woody-dicot clade, whereas *OsFAD2.1*–*OsFAD2.3* and *AtFAD2* formed separate branches. Bootstrap analysis revealed that most subfamily-level nodes received high support (≥95%), indicating strong topological robustness under data resampling and confirming the reliability of the subfamily classification. In contrast, some deeper branching patterns within subfamilies exhibited moderate-to-low bootstrap support (<70%), suggesting a weaker phylogenetic signal or potential site conflicts at those nodes; such relationships should be interpreted with caution and warrant further validation using alternative reconstruction methods or additional sequence data. Overall, the phylogenetic analysis not only confirmed the conserved subfamily classification of the *FAD* gene family across angiosperms, but also revealed clear lineage-specific clustering patterns that reflect the closer evolutionary relationships among woody dicot species.

### 2.3. Conserved Motif, Domain, and Gene Structure Analysis of JrFAD Proteins

**Figure 2:**
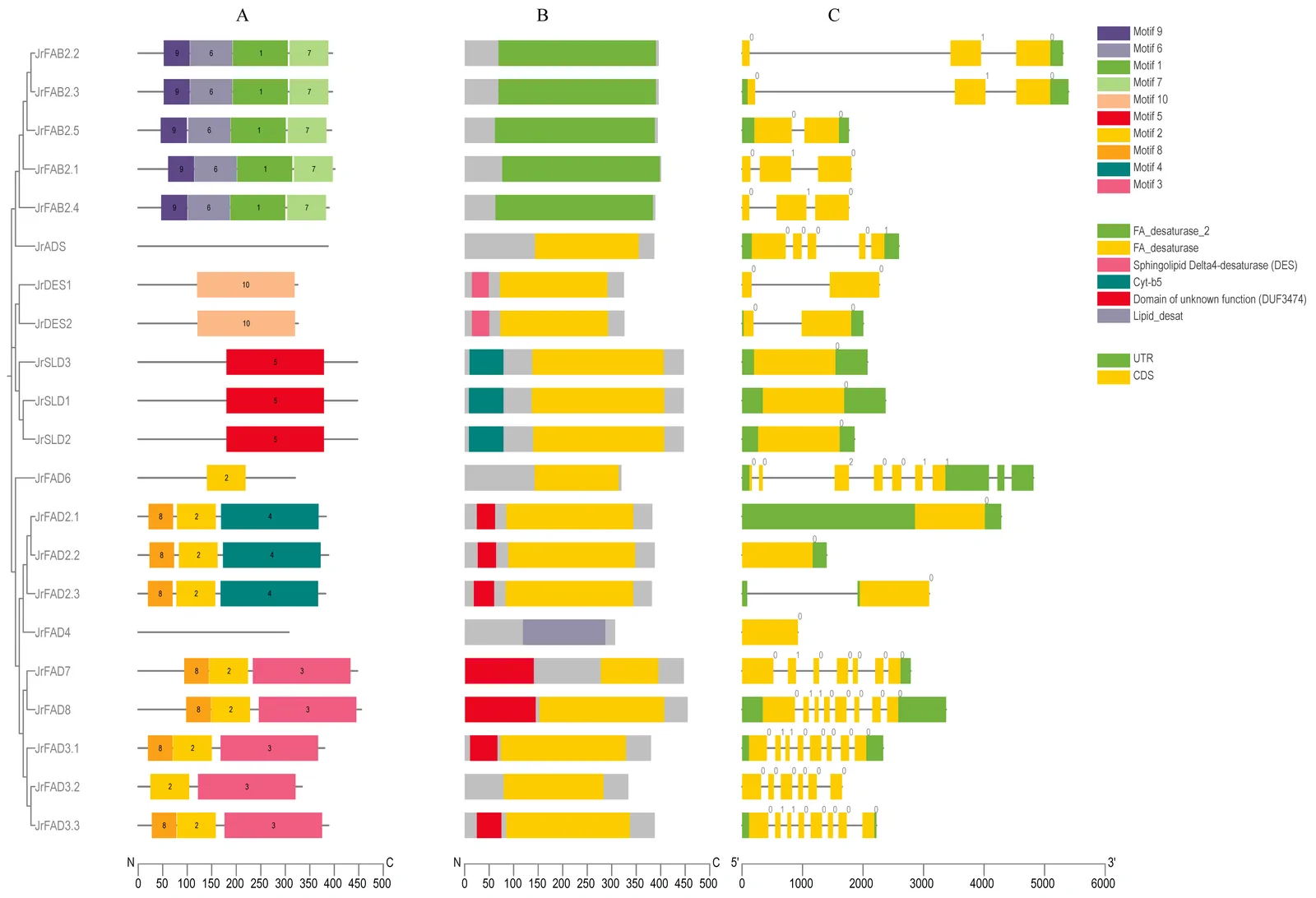
Conserved motif, conserved domain, and gene structure of the *FAD* gene family in *Juglans regia*. (**A**) The phylogenetic tree (**left**) constructed based on full-length amino acid sequences, and the distribution of conserved motifs (**right**) identified by MEME analysis. Each motif is represented by a distinct colored box, with its position corresponding to the relative location within the protein sequence. (**B**) Conserved domain architecture annotated using Pfam and CDD databases. Color-coded boxes represent distinct domain types. (**C**) Gene structure diagrams displaying coding sequences (CDS, yellow boxes), untranslated regions (UTRs, green boxes), and introns (black lines). 0, 1, 2 at the 5′ intron boundary represents intron splicing after the third, the first and the second of the codon, respectively.

To further compare the structural characteristics of *JrFAD* members, conserved motif and gene structure were analyzed jointly. Members within the same subfamily generally showed similar motif compositions and gene structures, whereas clear differences were observed among different subfamilies (Figure 2A). All five *FAB2* subfamily members contained Motif 9, Motif 6, Motif 1, and Motif 7, indicating a relatively conserved protein structure within this subfamily. In the *ADS/SLD/DES* subfamily, all *DES* members contained Motif 10, while all *SLD* members contained Motif 5, and members within each group showed similar gene structures. In the *FAD2/FAD6* (ω-6) subfamily, all *FAD2* members contained Motif 8, Motif 2, and Motif 4, and each of them contained only one exon. In contrast, *JrFAD6* contained only Motif 2. In the *FAD3/FAD7/FAD8* (ω-3) subfamily, all members except *JrFAD3.2* contained Motif 8, Motif 2, and Motif 3. Members of this subfamily also possessed more exons and showed relatively consistent gene structures. No obvious conserved motifs were predicted in *JrADS* or *JrFAD4*, which may be related to their subfamily-specific domain characteristics. *JrADS* belongs to the *ADS* subfamily, while *JrFAD4* belongs to the *FAD4* subfamily containing the TMEM189 domain. These two proteins may have relatively low sequence conservation compared with typical FAD proteins. Overall, the conserved motif composition and gene structure of *JrFAD* family members were largely consistent with their phylogenetic classification, further supporting the reliability of the subfamily division.

Conserved domain analysis further highlighted the differences among the subfamilies (Figure 2B). All five members of the *FAB2* subfamily specifically contained the FA_desaturase_2 domain. Within the *ADS/SLD/DES* subfamily, the *JrSLD1–JrSLD3* members simultaneously harbored the Cyt-b5 and FA_desaturase domains, whereas *JrDES1* and *JrDES2* contained the unique Sphingolipid Delta4-desaturase (DES) domain, and *JrADS* possessed only the FA_desaturase domain. Most members of the *FAD2* subfamily and the *FAD3/FAD7/FAD8* subfamily (except *JrFAD6*) contained both the FA_desaturase and domain of unknown function (DUF3474) domains, suggesting a potential evolutionary association in domain composition between these two subfamilies; however, *JrFAD6* contained only the FA_desaturase domain and lacked DUF3474. Notably, *JrFAD4* was the sole member that harbored the Lipid_desat domain. Beyond the differences in conserved domain composition, we further investigated the structural divergence at the gene level by analyzing intron splicing phases. Three intron splicing phases were observed in the walnut *FAD* gene family: phase 0 (splicing after the third nucleotide of the codon), phase 1 (splicing after the first nucleotide of the codon), and phase 2 (splicing after the second nucleotide) [25]. Most genes in this family possess phases 0 and 1, whereas the *JrFAD6* gene contains all three intron splicing phases (Figure 2C). In the *ADS/DES/SLD*, *FAD2*, and *FAD4* subfamilies, most members contain only phase 0 introns, whereas most members in the *FAB2* and *FAD3/FAD7/FAD8* subfamilies contain both phase 0 and phase 1 introns.

To further characterize the sequence features of *JrFAD* conserved motifs, sequence logo analysis was performed for the 10 identified motifs (Figure S2). The results showed that members within the same subfamily shared similar motif compositions, and several key amino acid residues were highly conserved. In particular, two conserved histidine motifs, EENRH and DEKRH, were identified in Motif 6 and Motif 1, respectively, both of which were shared by *FAB2* subfamily members. These conserved histidine motifs are characteristic features of the soluble *FAD* (*FAB2/SAD*) family. These results further indicate that JrFAD proteins are structurally conserved within subfamilies.

### 2.4. Chromosomal Distribution of JrFAD Genes

**Figure 3:**
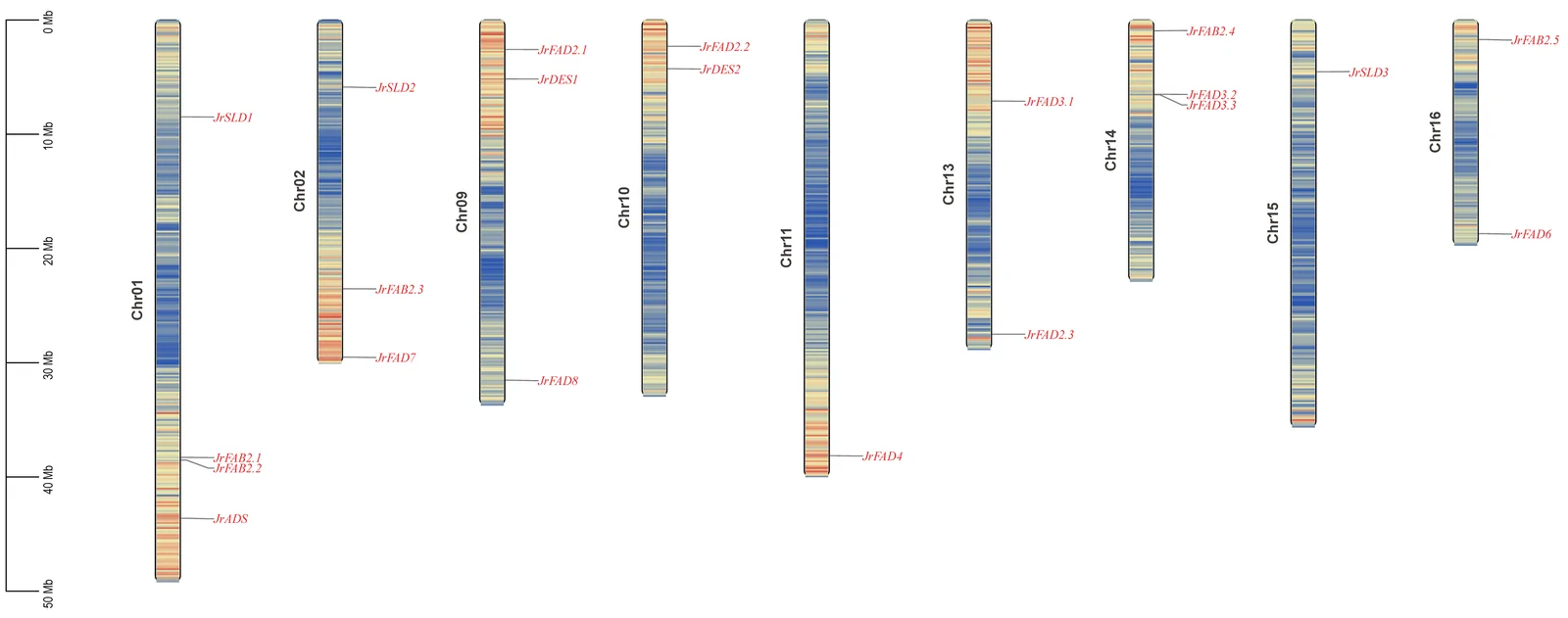
Chromosomal mapping of *FAD* gene family members in *Juglans regia*. The physical locations of the 21 *JrFAD* family members are mapped onto chromosomes based on the *Juglans regia* genome assembly. Gene names are indicated in red and positioned according to their relative physical coordinates (Mb scale is shown on the left). The chromosome lengths are represented proportionally, with red in the vertical bars along each chromosome indicating regions of greater gene density.

Based on walnut genome annotation information, the chromosomal distribution of the 21 *JrFAD* genes was analyzed. The results showed that *JrFAD* genes were unevenly distributed across nine chromosomes (Figure 3). Chr01 contained the largest number of *JrFAD* genes, with four members. Chr02, Chr09, and Chr14 each contained three *JrFAD* genes; Chr10, Chr13, and Chr16 each contained two members; and Chr11 and Chr15 each contained only one member. Notably, *JrFAD3.2* and *JrFAD3.3* were located adjacent to each other on Chr14, showing a clear tandem duplication pattern. This suggests that local tandem duplication may have contributed to the formation of some *FAD3* subfamily members. However, most *JrFAD* genes were dispersed across different chromosomes, indicating that the expansion of the *JrFAD* gene family may have been shaped by multiple duplication mechanisms.

### 2.5. Cis-Acting Element Analysis of JrFAD Gene Promoters

**Figure 4:**
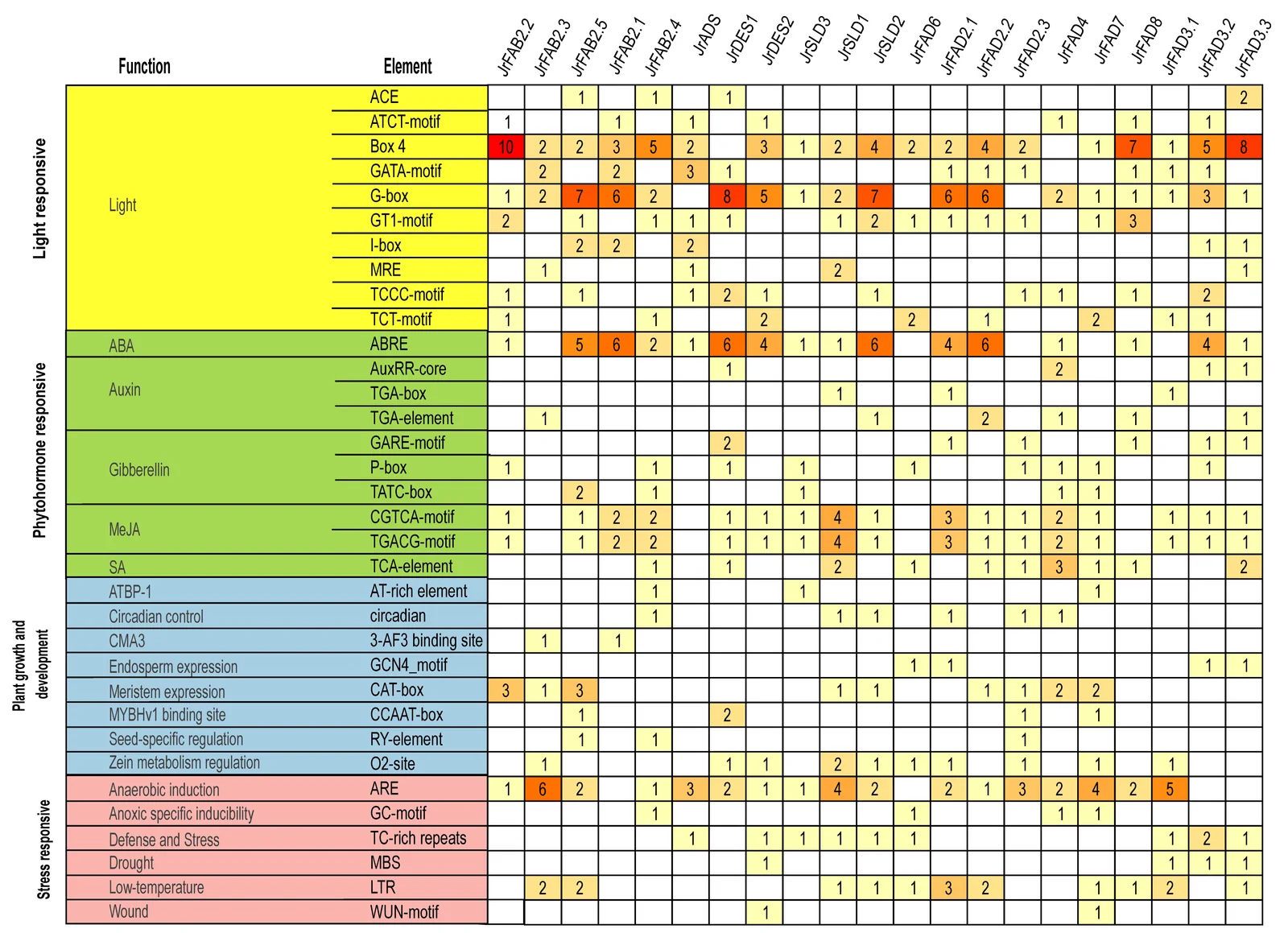
Promoter *cis*-acting element distribution analysis of *FAD* gene family members from *Juglans regia*. Elements are grouped into four functional categories, distinguished by color: light-responsive elements (yellow), phytohormone-responsive elements (green), plant growth- and development-related elements (blue), and stress-responsive elements (red). The intensity of the color reflects the abundance of each element within the corresponding promoter.

To investigate the potential transcriptional regulatory mechanisms of *JrFAD* genes, *cis*-acting elements were predicted in the 2000 bp upstream promoter regions of each member. The results showed that the promoter regions of *JrFAD* genes contained diverse regulatory elements, including light-responsive elements, phytohormone-responsive elements, stress-responsive elements, and plant growth- and development-related elements (Figure 4). Light-responsive elements were the most widely distributed in the *JrFAD* promoters. Among them, Box 4 showed the highest frequency, with 66 elements identified in all members except *JrDES1* and *JrFAD4*. A total of 62 G-box elements were detected and were present in all members except *JrADS*, *JrFAD6*, and *JrFAD2.3*. Among the phytohormone-responsive elements, the methyl jasmonate-responsive CGTCA motif and TGACG motif, together with the abscisic acid-responsive ABRE element, were highly abundant, each reaching 50 elements. MeJA-responsive elements were found in the promoters of 17 *JrFAD* genes, suggesting that *JrFAD* expression may be broadly regulated by phytohormone signaling. Among the stress-responsive elements, the anaerobic induction-related ARE element was the most abundant, with 42 elements identified in the promoters of 17 *JrFAD* genes. *JrFAB2.3* contained the highest number of ARE elements, with six copies. A total of 17 low-temperature-responsive LTR elements were also detected. Among the plant growth- and development-related elements, the meristem expression-related CAT-box was the most frequent, with 15 elements identified. In addition, circadian rhythm-related elements, O2-site elements associated with zein metabolism regulation, and seed-specific RY elements were also detected. These results indicate that *JrFAD* gene expression could potentially be regulated by light, hormones, stress, and developmental signals, although this remains a prediction requiring experimental confirmation.

### 2.6. Duplication Events and Collinearity Analysis of the JrFAD Gene Family

**Figure 5:**
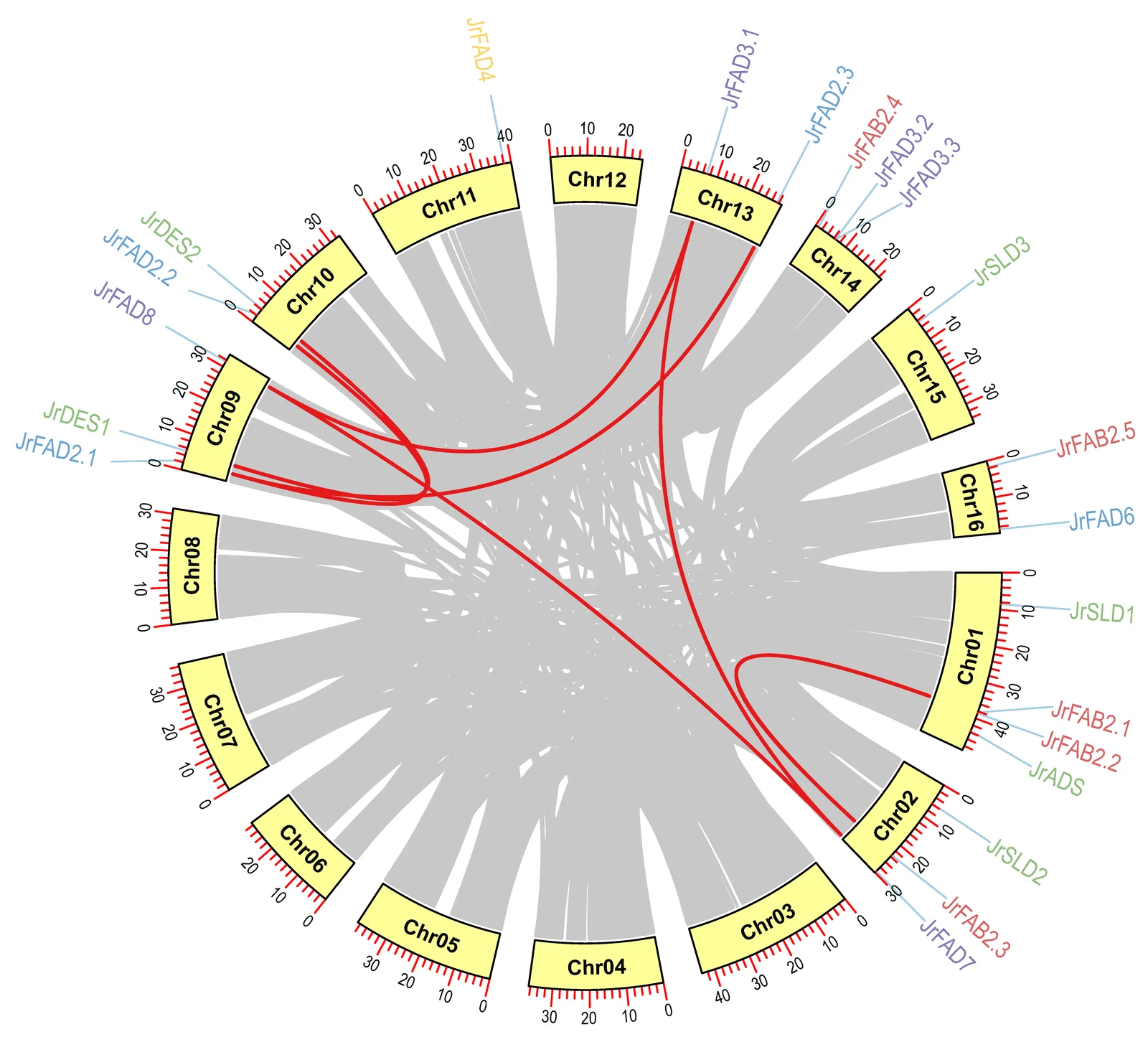
Collinearity analysis of *FAD* gene family members from *Juglans regia*. Chromosomes are represented by orange boxes. Duplicated gene pairs are connected with red lines.

To explore the expansion mechanism of the *JrFAD* gene family, duplication types and intraspecific collinearity relationships were analyzed for the 21 *JrFAD* genes. Duplication type analysis revealed that the *JrFAD* gene family included multiple duplication modes, including WGD/segmental duplication, tandem duplication, dispersed duplication, and singleton genes (Table S4). Among them, WGD or segmental duplication represented the most frequent type, involving 10 members. Three members, *JrFAB2.4*, *JrFAD3.2*, and *JrFAD3.3*, were identified as tandem duplicated genes. Six members were classified as dispersed duplicated genes, whereas *JrFAD4* and *JrFAD6* were single-copy genes. These results suggest that WGD or segmental duplication may have been the major driving force underlying *JrFAD* gene family expansion in the walnut. Intraspecific collinearity analysis identified seven duplicated gene pairs with significant collinearity relationships (Figure 5). The *FAD3/FAD7/FAD8* (ω-3) subfamily contained the largest number of duplicated gene pairs, including *JrFAD3.1/JrFAD7*, *JrFAD3.1/JrFAD8*, and *JrFAD7/JrFAD8*. The *FAD2/FAD6* (ω-6) subfamily contained two duplicated gene pairs, *JrFAD2.1/JrFAD2.2* and *JrFAD2.1/JrFAD2.3*. The *FAB2* and *ADS/SLD/DES* subfamilies each contained one duplicated gene pair, namely *JrFAB2.2/JrFAB2.3* and *JrDES1/JrDES2*, respectively.

**Figure 6:**
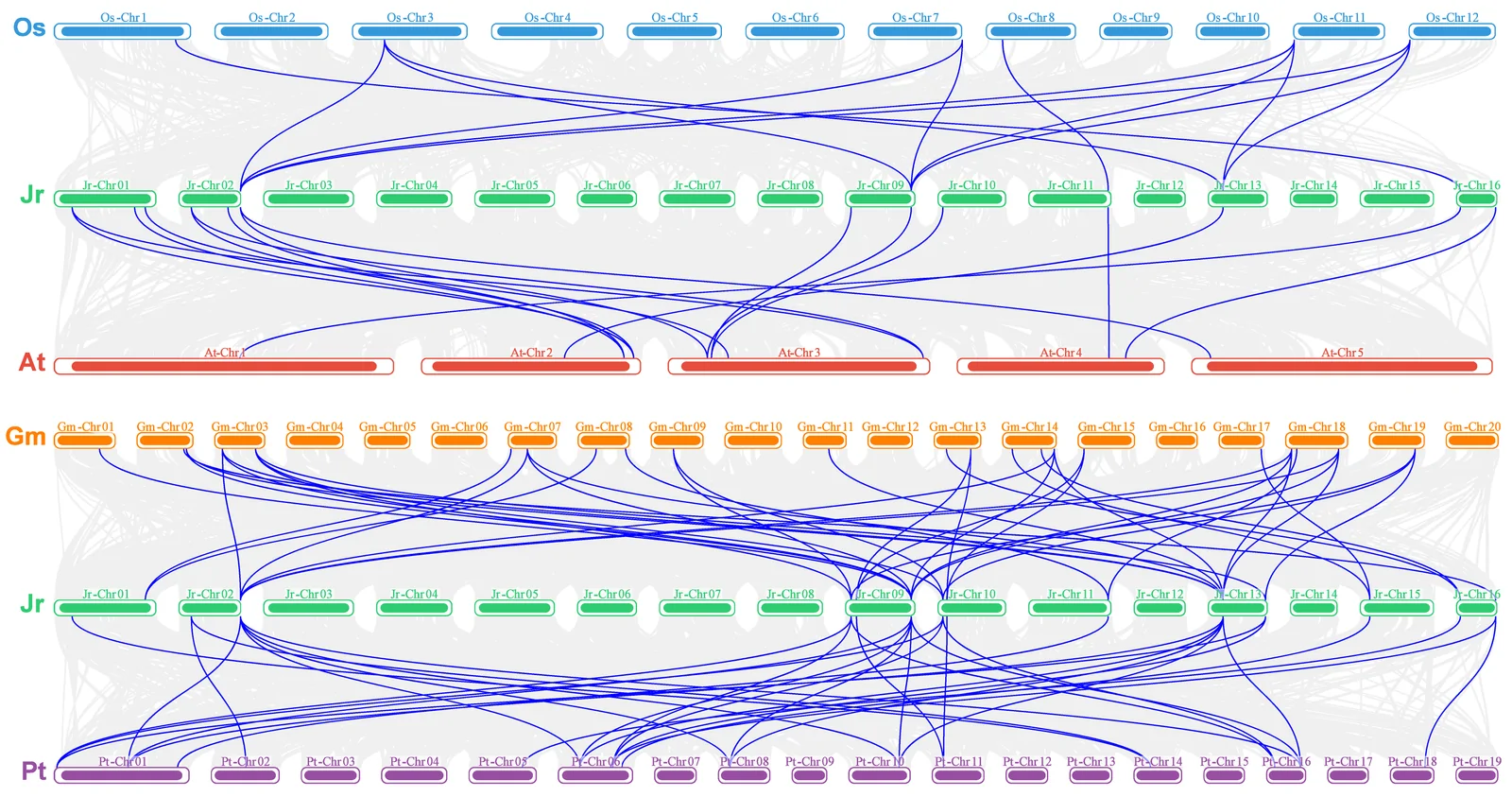
Interspecific collinearity analysis of *FAD* gene family members between *Juglans regia* and *Arabidopsis thaliana*, *Oryza sativa*, *Glycine max*, and *Populus trichocarpa*. Blue lines indicate orthologous gene pairs.

The Ka/Ks ratios of duplicated gene pairs were further calculated to evaluate selective pressure during evolution. The Ka/Ks values of all seven *JrFAD* duplicated gene pairs were less than 1, ranging from 0.0958 to 0.1471 (Table S5), indicating that these duplicated genes mainly experienced purifying selection and that their protein-coding sequences were under strong evolutionary constraints. To further assess the evolutionary conservation of the *JrFAD* gene family, interspecific collinearity relationships were compared between walnut and Arabidopsis, rice, soybean (*Glycine max*), and black cottonwood (*Populus trichocarpa*). The syntenic relationships between *JrFAD* genes and their homologs in other species are listed in Table S6. *JrFAD* genes showed varying degrees of orthologous collinearity with *FAD* genes from all four reference species (Figure 6). The strongest collinearity was observed between walnut and soybean, with 40 orthologous collinear gene pairs identified. Walnut also showed high collinearity with black cottonwood, with 33 orthologous collinear gene pairs detected. In contrast, 16 and 13 orthologous collinear gene pairs were identified between walnut and Arabidopsis and between walnut and rice, respectively. These results indicate that the *JrFAD* gene family is evolutionarily conserved in plants and that *FAD* genes in the walnut show closer collinear relationships with those in soybean and black cottonwood.

### 2.7. Expression Profiles and Gene–Metabolite Associations of the JrFAD Gene Family

**Figure 7:**
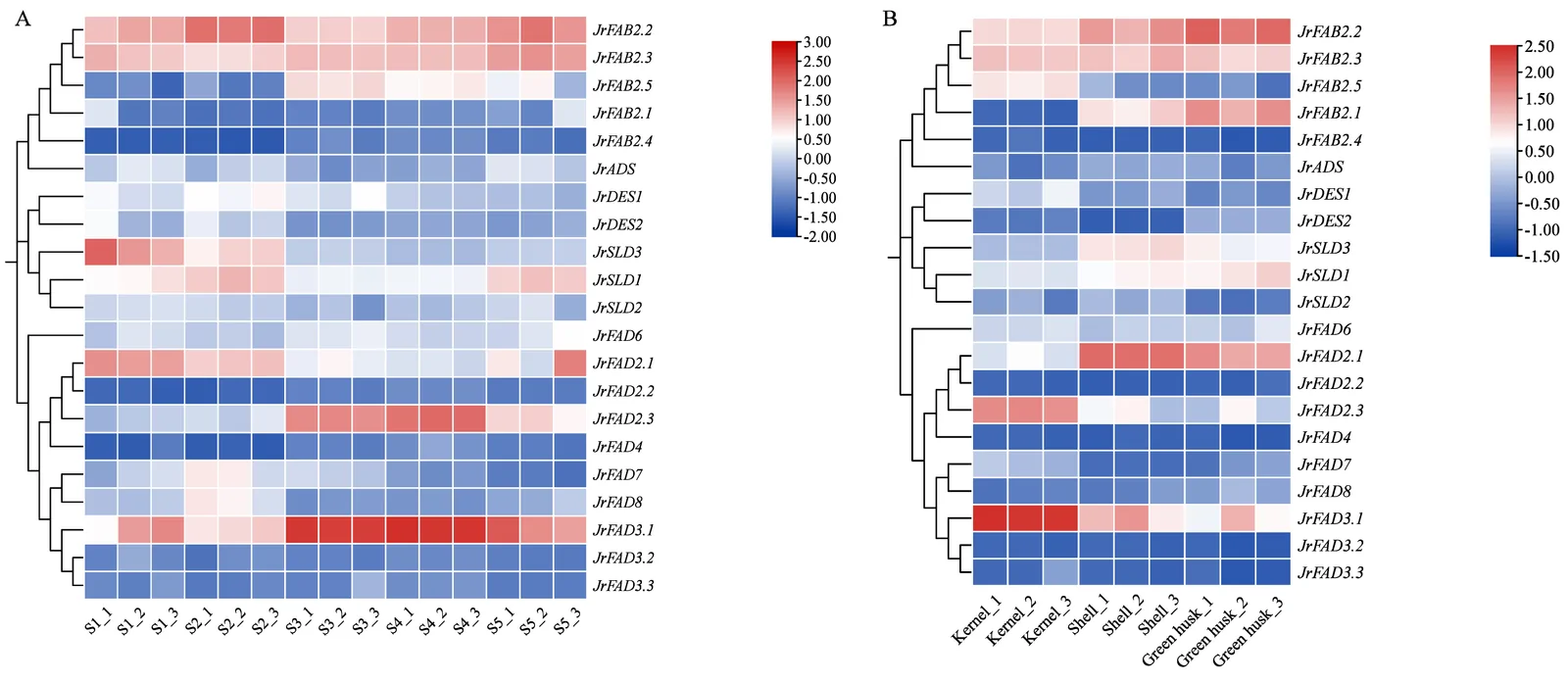
Expression pattern analysis of the *FAD* gene family in *Juglans regia*. (**A**) Heatmap showing expression levels in kernels at different developmental stages. (**B**) Heatmap showing expression levels in various tissues. The heatmap was drawn using TBtools-II v2.485 software based on the log2(FPKM) values.

To investigate the potential roles of *JrFAD* genes during walnut kernel development, transcriptome data were used to analyze the expression patterns of the 21 *JrFAD* genes across different kernel developmental stages (Table S7). The results showed that *JrFAD* family members displayed distinct expression patterns during kernel development (Figure 7A). Among them, *JrSLD1* and *JrFAB2.2* maintained relatively stable moderate expression levels throughout the developmental process, suggesting that they may participate in basal lipid metabolism. *JrFAD3.1* and *JrFAD2.3* exhibited clear stage-specific expression patterns during kernel development. In particular, *JrFAD3.1* showed a developmentally dynamic expression pattern, with relatively low expression at the early stages, elevated expression during the middle developmental period, and a subsequent decline at the late stage. This gene showed a typical increase-then-decrease expression pattern and was highly expressed during the rapid oil-accumulation period, supporting its prioritization as a candidate gene potentially associated with walnut kernel lipid metabolism in walnut kernels.

To systematically characterize gene co-expression patterns during walnut kernel development, weighted gene co-expression network analysis (WGCNA) was performed using 21,586 expressed genes from 15 samples representing five developmental stages. The cyan module contained 350 genes and was significantly positively correlated with S5 (r = 0.76, *p* = 0.001). GO enrichment analysis showed that genes in the cyan module were mainly associated with seed oil-body biogenesis, lipid storage, lipid localization, lipid droplets, seed development, and seed maturation (Figure S3). Nevertheless, the two candidate genes *JrFAD2.3* and *JrFAD3.1* showed peak expression at S3–S4, whereas the cyan module exhibited the strongest correlation with S5, indicating that the module-level pattern reflects late maturation processes rather than the individual dynamics of these two genes. Accordingly, the co-expression network provides supportive, but not definitive, evidence for their involvement in oil accumulation. The local co-expression network further showed that *JrFAD2.3* and *JrFAD3.1* were connected with the transcription factors *JrABI3-1* and *JrWRI1-1*; the fatty acid biosynthesis-related genes *JrKASI*, *JrKASII* and *JrKAR*; several *JrOle* genes; and *Jr11S* seed storage protein genes (Figure S3D). These results suggest that *JrFAD2.3* and *JrFAD3.1* may participate in a coordinated co-expression network associated with fatty acid metabolism, oil-body formation, and kernel maturation.

Five lipid-related features, putatively annotated as palmitic acid, stearic acid, oleic acid, a C18:2 fatty-acid feature, and a C18:3 fatty-acid feature, were selected from the negative-ion-mode LC–MS dataset for developmental profiling. The palmitic-acid and C18:2 fatty-acid features showed their highest mean signal intensities at S3, whereas the stearic-acid feature increased markedly at S4 and remained relatively high at S5. The oleic-acid feature maintained relatively high signal intensities during S1–S3, followed by a pronounced decrease at S4 and a partial recovery at S5. The C18:3 fatty-acid feature showed its highest mean signal intensity at S2 and decreased substantially during S3–S5. The individual signal intensities for the three biological replicates at each stage are provided in Table S8. Importantly, these values represent relative signal intensities of extractable fatty-acid-related features detected by untargeted negative-ion LC–MS rather than the fatty-acid composition of kernel storage lipids.

To further relate *JrFAD* expression to developmental changes in lipid-related LC–MS features, Pearson correlation analysis was performed between the 21 *JrFAD* genes and five lipid-related features across the same 15 kernel samples, resulting in 105 gene–feature tests. After Benjamini–Hochberg correction for multiple testing, three associations remained significant at FDR < 0.05: *JrSLD3* showed positive developmental covariation with the putatively annotated C18:3 fatty-acid feature (r = 0.782, *p* = 5.74 × 10^−4^, FDR = 0.0443), whereas *JrFAD2.1* (r = −0.762, *p* = 9.60 × 10^−4^, FDR = 0.0443) and *JrFAD3.2* (r = −0.750, *p* = 1.27 × 10^−3^, FDR = 0.0443) showed inverse developmental covariation with the stearic-acid feature (Figure S4; Table S9). The complete r, raw *p*, and FDR-adjusted *p* values for all 105 tests are provided in Table S9. These statistical associations, especially the inverse covariation with stearate, should be interpreted as correlative patterns rather than direct enzymatic relationships. Notably, this distinct set of correlated genes represents statistical associations rather than confirmatory evidence for the primary candidates, offering testable hypotheses for future functional studies.

The expression patterns of the 21 *JrFAD* genes were further compared among kernel, husk, and shell tissues (Table S10). *JrFAD* family members also showed clear tissue-specific expression differences (Figure 7B). *JrFAD3.1* and *JrFAD2.3* were highly expressed in kernels but showed low expression levels in husk and shell tissues. For example, the mean FPKM value of *JrFAD3.1* in kernels reached 4809.35, whereas its mean FPKM values in husk and shell tissues were only 38.48 and 65.39, respectively, accounting for less than 1/70 of the expression level in kernels. This result indicates that *JrFAD3.1* exhibits a strong kernel-preferential expression pattern. In contrast, *JrFAB2.1* and *JrFAD2.1* showed relatively high expression levels in husk and shell tissues, suggesting that they may be involved in the development of external fruit tissues or environmental responses. *JrFAB2.2* maintained relatively high expression levels in kernel, husk, and shell tissues, showing a constitutive expression pattern and suggesting its potential role in basal lipid metabolism across different walnut tissues.

To better illustrate the expression dynamics of key candidate genes, the expression levels of *JrFAD3.1* and *JrFAD2.3* were further analyzed during kernel development (Figure S5; Table S11). Both *JrFAD3.1* and *JrFAD2.3* maintained high expression levels from 80 to 100 DAF, but their expression peaks occurred at different times. *JrFAD3.1* reached its peak expression at 87 DAF, whereas *JrFAD2.3* peaked at 100 DAF. In addition, the overall expression level of *JrFAD3.1* was markedly higher than that of *JrFAD2.3*. As 80–100 DAF corresponds to the key period of rapid oil accumulation in walnut kernels, the elevated transcript abundance of *JrFAD3.1* and *JrFAD2.3* during this period supports their prioritization as developmental candidate genes potentially associated with unsaturated fatty acid metabolism are candidate genes potentially associated with unsaturated fatty-acid-related LC–MS features in walnut kernels.

## 3. Discussion

### 3.1. Evolutionary Characteristics and Expansion Mechanisms of the JrFAD Gene Family

Fatty acid desaturases are essential enzymes involved in fatty acid metabolism, membrane lipid homeostasis, and stress adaptation in plants [6,7]. With the increasing availability of plant genome resources, *FAD* gene families have been identified in many species, but their family sizes vary considerably. For instance, 20 *FAD* genes have been reported in rice [26], 29 in soybean [27,28], 26 in tomato (*Solanum lycopersicum*) [5], 34 in pear (*Pyrus bretschneideri*) [29] and 68 in common wheat [30]. In this study, 21 *JrFAD* genes were identified in the walnut, a number close to that in rice but smaller than those in soybean and wheat. This difference suggests that the expansion of the *FAD* gene family is species-specific and may be associated with distinct genome duplication and evolutionary histories.

Phylogenetic analysis classified the 21 JrFAD proteins, together with FAD proteins from Arabidopsis, rice, black cottonwood, and hickory, into five subfamilies: *FAB2*, *FAD4*, *ADS/SLD/DES*, *FAD2/FAD6*, and *FAD3/FAD7/FAD8*. This classification is consistent with previous studies in apples (*Malus domestica*) [31], bananas (*Musa × paradisiaca*) [32], and wheat [30], indicating that *FAD* genes are evolutionarily conserved in plants. The clustering pattern also reflects their potential functional differentiation: *FAB2* members are mainly related to the conversion of stearic acid to oleic acid, *FAD2/FAD6* members participate in linoleic acid biosynthesis, and *FAD3/FAD7/FAD8* members are associated with α-linolenic acid formation [6,12,33]. Conserved motif, domain, and gene structure analyses further supported this phylogenetic classification. *JrFAD* members within the same subfamily generally shared similar motif compositions, domain organizations, and exon–intron structures, whereas clear structural differences were observed among different subfamilies [7,31,34]. For example, *FAB2* members contained conserved histidine motifs, including EENRH and DEKRH, which are typical structural features of soluble *FAD/SAD* proteins [35]. *FAD2* members showed a relatively simple single-exon structure, whereas *FAD3/FAD7/FAD8* members usually contained more exons, suggesting that different subfamilies may have experienced different degrees of structural conservation and functional divergence. Chromosomal localization showed that the 21 *JrFAD* genes were unevenly distributed on nine walnut chromosomes, a pattern commonly observed in plant *FAD* gene families [7,36]. *JrFAD3.2* and *JrFAD3.3* were adjacent on Chr14, indicating that tandem duplication contributed to the formation of some *FAD3* members. However, duplication type and collinearity analyses showed that WGD/segmental duplication was the major contributor to *JrFAD* family expansion, involving 10 members, whereas tandem duplication contributed to only a limited number of genes. Seven duplicated *JrFAD* gene pairs were identified, and the *FAD3/FAD7/FAD8* subfamily contained the largest number of duplicated pairs, suggesting that the ω-3 fatty acid desaturase subfamily played an important role in the evolutionary expansion of walnut *FAD* genes.

All duplicated *JrFAD* gene pairs had Ka/Ks values below 1, ranging from 0.0958 to 0.1471, indicating that these genes have mainly undergone purifying selection during evolution [30]. This suggests that their protein-coding sequences have been constrained and that their functions may have been largely conserved. Interspecific collinearity analysis revealed more collinear gene pairs between walnut and soybean or black cottonwood than between walnut and Arabidopsis or rice, indicating that *JrFAD* genes share stronger evolutionary conservation with those of closely related dicotyledonous species. Overall, these results suggest that the *JrFAD* gene family is conserved in plants, while its expansion and diversification in the walnut were mainly shaped by WGD/segmental duplication and purifying selection.

### 3.2. Expression Regulation and Potential Functional Analysis of JrFAD Genes

*Cis*-acting elements are important regulatory regions for transcription factor binding and play key roles in tissue-specific expression, developmental regulation, and stress responses [37,38]. In this study, abundant light-, hormone-, stress-, and development-related elements were identified in the 2000 bp upstream promoter regions of *JrFAD* genes, suggesting that *JrFAD* expression may be regulated by multiple signaling pathways [39]. Among them, light-responsive elements were the most widely distributed, with Box 4 and G-box occurring at high frequencies, indicating that light signaling may be involved in the transcriptional regulation of walnut *FAD* genes. Similar enrichment of light-responsive elements has also been reported in *FAD* gene family studies of tea/oil tea and potato, implying close relationships between *FAD* genes, plastid function, and membrane lipid metabolism [7,12].

Hormone- and stress-responsive elements were also widely present in the *JrFAD* promoters. In particular, MeJA-responsive CGTCA motifs and TGACG motifs, ABA-responsive ABRE elements, anaerobic induction-related ARE elements, and low-temperature-responsive LTR elements were frequently detected. MeJA and ABA are important signaling molecules involved in plant development, seed maturation, and stress responses; therefore, the enrichment of these elements suggests that *JrFAD* genes may participate not only in walnut kernel oil accumulation but also in environmental adaptation. This is consistent with previous studies showing that *FAD* genes contribute to stress tolerance by modulating fatty acid unsaturation and maintaining membrane fluidity and stability [6,7,40,41]. Nevertheless, the regulatory implications drawn from these promoter analyses should be regarded as preliminary hypotheses that require further experimental validation through approaches such as stress treatment combined with expression profiling, promoter–reporter assays, or transcription factor binding experiments. Expression pattern analysis further indicated functional differentiation among *JrFAD* family members. During walnut kernel development, several *JrFAD* genes showed dynamic expression changes, suggesting their potential roles in oil accumulation and relative LC–MS signal intensity regulation. Among them, *JrSLD1* and *JrFAB2.2* maintained relatively stable expression levels, implying possible roles in basal lipid metabolism or membrane lipid homeostasis. In contrast, *JrFAD3.1* and *JrFAD2.3* exhibited clear stage- and tissue-specific expression patterns and were highly expressed during the rapid oil-accumulation period of walnut kernels.

Notably, *JrFAD3.1* showed a strong kernel-preferential expression pattern. *JrFAD3.1* showed relatively low transcript abundance during the early developmental stages. Consistent with the corrected DESeq2 analysis, no significant difference was detected between S1 and S2, whereas its expression increased markedly at S3, reached a high level during the subsequent rapid oil-accumulation period, and declined substantially at S5. Tissue expression analysis showed that the average FPKM value of *JrFAD3.1* in kernels reached 4809.35, more than 70-fold higher than that in husk and shell tissues. Since *FAD3* belongs to the ω-3 fatty acid desaturase family and mainly catalyzes the conversion of linoleic acid (C18:2) to α-linolenic acid (C18:3), the high expression of *JrFAD3.1* during kernel oil accumulation strongly suggests that it may be involved in α-linolenic acid biosynthesis in walnut kernels. *JrFAD2.3* also showed high expression in kernels during the rapid oil accumulation stage, although its expression peak occurred later than that of *JrFAD3.1*.

The developmental expression patterns of *JrFAD2.3* and *JrFAD3.1* are broadly consistent with the established biochemical functions of FAD2- and FAD3-type desaturases, respectively. Therefore, *JrFAD2.3* and *JrFAD3.1* may act sequentially in the formation of polyunsaturated fatty acids in walnut kernels [6,12,42,43].

Gene and metabolite correlation analysis identified several additional *JrFAD* members associated with variation in the relative abundances of lipid-related metabolites. These associations are statistical and should be regarded as exploratory evidence rather than experimental validation of gene function. In addition, some *JrFAD* genes displayed higher expression in non-kernel tissues. For example, *JrFAB2.1* and *JrFAD2.1* were preferentially expressed in husk and shell tissues, suggesting that they may be involved in membrane lipid metabolism, tissue development, or environmental responses in external fruit tissues. *JrFAB2.2* showed relatively broad expression across kernel, husk, and shell tissues, indicating a possible conserved role in basal fatty acid metabolism. Taken together, these results suggest that *JrFAD* genes have undergone functional differentiation among different tissues and developmental stages. In particular, *JrFAD3.1* and *JrFAD2.3* are potential candidate genes for regulating polyunsaturated fatty acid biosynthesis during walnut kernel oil accumulation and provide valuable targets for future functional validation and walnut oil quality improvement [44]. A limitation of the present study is that JrFAD protein abundance and enzymatic activity were not experimentally determined. In addition, the STRING-derived network represents homolog-based computational predictions rather than experimentally verified physical interactions in the walnut. Future studies should employ validated member-specific antibodies, immunoblotting, targeted proteomics, subcellular localization assays, and biochemical activity measurements to examine JrFAD proteins across developmental stages, tissues, and defined stress treatments. Collectively, the integrated evidence from developmental expression, WGCNA, gene–metabolite associations, and predicted protein interactions further supports the potential involvement of these candidate *JrFAD* genes in walnut kernel fatty acid metabolism (Figure S6). The potential roles of other *JrFAD* genes identified through transcriptome profiling, WGCNA, or gene–metabolite correlations remain predictive and require further experimental validation.

## 4. Materials and Methods

### 4.1. Plant Materials

The walnut cultivar ‘Liaoning 1’ was used as the plant material in this study. Samples were collected from the Xi’an Botanical Garden. According to the developmental process of walnut kernels, the kernel, green husk, and shell tissues were collected at different developmental stages. The five main stages S1–S5 correspond to 40, 60, 80, 100, and 120 days after flowering (DAF), respectively. Additional intermediate time points (50, 67, 74, 87, 94, 110 DAF) were also sampled for transcriptome analysis to achieve higher temporal resolution. Three biological replicates were prepared for each tissue and developmental stage, and each replicate consisted of samples collected from three plants with similar growth status and no obvious symptoms of pests or diseases. All collected samples were immediately frozen in liquid nitrogen and stored at −80 °C for subsequent gene expression analysis.

### 4.2. Identification of JrFAD Gvene Family Members in Walnut

The genome assembly used for the genome-wide identification of the *JrFAD* gene family was the JRELIAO1 reference assembly generated from the walnut cultivar ‘Liaoning 1’. The exact genome assembly FASTA file and the corresponding complete genome-wide GFF3 annotation used in this study are publicly available in Figshare under DOI 10.6084/m9.figshare.33327072 and DOI 10.6084/m9.figshare.33350970, respectively. The JRELIAO1 assembly has a total assembled size of 550.89 Mb, with a contig N50 of 35.68 Mb and a scaffold N50 of 35.68 Mb. The GC content was 36.45%, and BUSCO analysis indicated 98.9% completeness. A total of 35,294 genes were annotated in the genome. The predicted protein set used for BLASTP- and HMM-based *JrFAD* identification was derived from the JRELIAO1 genome annotation. All chromosome coordinates, gene structures, and other annotation information used in the subsequent analyses were obtained from the same JRELIAO1 assembly–annotation release.

The predicted protein set derived from the JRELIAO1 genome annotation has been deposited separately in Figshare under DOI 10.6084/m9.figshare.33384184. The FAD protein sequences of *Arabidopsis thaliana* were used as query sequences, and the corresponding sequence information was obtained from the TAIR database (https://www.arabidopsis.org/) [45]. First, BLASTP was used to search the JRELIAO1 predicted protein set for homologous sequences, with an E-value threshold of ≤1 × 10^−5^. Subsequently, hidden Markov model (HMM) profiles of fatty acid desaturase-related conserved domains, including the FA_desaturase domain (PF00487), FA_desaturase_2 domain (PF03405), and TMEM189 domain (PF10520), were downloaded from the Pfam database (http://pfam.xfam.org/) [46]. Domain searches were then performed against the JRELIAO1 predicted protein set using the Simple HMM Search tool in TBtools-II v2.485, with an E-value threshold of ≤1 × 10^−5^ [47]. The results obtained from BLASTP and HMM searches were merged, and redundant sequences were removed to obtain candidate JrFAD proteins. All candidate proteins were further verified using the NCBI Conserved Domain Database (CDD; https://www.ncbi.nlm.nih.gov/cdd/). Sequences lacking typical *FAD* conserved domains were removed, and the remaining candidates were identified as members of the walnut *JrFAD* gene family [48]. Finally, the *JrFAD* genes were systematically renamed according to their physical positions on chromosomes.

### 4.3. Physicochemical Properties and Subcellular Localization Prediction

The physicochemical properties of the identified JrFAD proteins, including amino acid length, molecular weight, theoretical isoelectric point, instability index, aliphatic index, and grand average of hydropathicity, were predicted using the ExPASy ProtParam online tool (https://web.expasy.org/protparam/) [49]. Subcellular localization prediction was performed using the CELLO v.2.5 online server (https://cello.life.nctu.edu.tw/) [50]. These tools provide computational predictions based on sequence features; experimental validation is required to confirm actual protein localization. Secondary structure prediction was completed using the online tool SOPMA (https://npsa.lyon.inserm.fr/cgi-bin/npsa_automat.pl?page=/NPSA/npsa_sopma.html) [51].

### 4.4. Phylogenetic Tree Construction

The phylogenetic relationships of walnut JrFAD proteins were examined using FAD protein sequences from walnut, *Arabidopsis*, rice, black cottonwood, and hickory. Multiple sequence alignment was performed using the MUSCLE algorithm implemented in MEGA 11.0. Based on the alignment results, a phylogenetic tree was constructed using the maximum likelihood (ML) method. Bootstrap analysis was performed with 1000 replicates to evaluate the reliability of each branch, and the remaining parameters were kept as default settings [52]. The resulting phylogenetic tree was visualized and edited using the iTOL online tool (https://itol.embl.de/) [53].

### 4.5. Conserved Motif, Domain, and Gene Structure Analysis

Conserved motifs in JrFAD protein sequences were analyzed using the MEME online program (https://meme-suite.org/meme/) [54]. The maximum number of motifs was set to 10, and the motif length was set from six to 200 amino acids. Other parameters were kept as default settings. Based on the complete JRELIAO1 genome annotation file (GFF3; Figshare DOI 10.6084/m9.figshare.33350970), the exon, intron, and CDS position information of *JrFAD* genes was extracted. The gene structures conserved motif, and domain distributions of *JrFAD* genes were then visualized using the Gene Structure View module in TBtools-II v2.485 [47].

### 4.6. Chromosomal Distribution and Duplication Analysis

The chromosome numbers and genomic coordinates of the *JrFAD* genes were extracted from the JRELIAO1 genome annotation file (GFF3; Figshare DOI 10.6084/m9.figshare.33350970). The Gene Location Visualize function in TBtools-II v2.485 was used to draw the chromosomal distribution map of *JrFAD* genes, thereby showing their distribution patterns on walnut chromosomes [47]. The expansion mechanism of the *JrFAD* gene family in the walnut was explored through intraspecific collinearity analysis using the One Step MCScanX tool in TBtools-II v2.485, with an E-value threshold of ≤1 × 10^−5^ [47]. The duplication types and intraspecific collinearity relationships of *JrFAD* genes were analyzed based on the MCScanX results and visualized using the Advanced Circos tool in TBtools-II v2.485.

### 4.7. Collinearity Analysis and Selection Pressure Estimation

For the duplicated gene pairs identified, the nonsynonymous substitution rate (Ka), synonymous substitution rate (Ks), and Ka/Ks ratio were calculated to evaluate the selection pressure acting on *JrFAD* genes during evolution. A Ka/Ks ratio > 1 indicates positive selection, Ka/Ks = 1 indicates neutral selection, and Ka/Ks < 1 indicates purifying selection [30]. The evolutionary conservation of the *JrFAD* gene family among different species was further assessed using four representative species, including *Arabidopsis thaliana*, rice, soybean, and black cottonwood. The genome sequences, protein sequences, and annotation files of these species were downloaded from the Phytozome database (https://phytozome-next.jgi.doe.gov/) [55]. The One Step MCScanX tool in TBtools-II v2.485 was used to perform collinearity analysis between walnut protein sequences and protein sequences from the four selected species, with an E-value threshold of ≤1 × 10^−5^. Finally, the Dual Synteny Plot tool in TBtools-II v2.485 was used to visualize the collinearity relationships between walnut and the other species, and the number of orthologous gene pairs involving *JrFAD* genes was counted [47].

### 4.8. Cis-Acting Element Prediction and Gene Expression Analysis

The 2000 bp upstream promoter sequences from the transcription start site of each *JrFAD* gene were extracted using TBtools-II v2.485 to investigate the potential transcriptional regulatory features of *JrFAD* genes [47]. The *cis*-acting elements in the promoter regions were predicted and annotated using the PlantCARE online database (http://bioinformatics.psb.ugent.be/webtools/plantcare/html/) [56]. Based on the prediction results, the distribution of different types of *cis*-acting elements was visualized using the HeatMap tool in TBtools-II v2.485 [47].

Expression pattern analysis of the *JrFAD* gene family was performed based on walnut transcriptome data to reveal their expression characteristics in different tissues and during kernel development. The transcriptome data were obtained from previous sequencing results generated in our laboratory and included different developmental stages of walnut kernels, as well as green husk and shell tissues.

Raw read counts from the 15 verified RNA-seq libraries were used as input for DESeq2 for count normalization and differential expression analysis. Four adjacent-stage comparisons were performed: S1 versus S2, S1 versus S4, S2 versus S3, S3 versus S4, and S4 versus S5. In the reported contrasts, positive log2FoldChange values indicate higher expression in the first stage of the comparison, whereas negative values indicate higher expression in the second stage. Genes with |log2FoldChange| ≥ 1 and Benjamini–Hochberg-adjusted *p* values (FDR) < 0.05 were classified as significantly differentially expressed. Complete raw counts and DESeq2 statistics are provided in Table S12.

According to the gene annotation information, the FPKM values of 21 *JrFAD* genes were extracted from the transcriptome expression matrix. Subsequently, the HeatMap tool in TBtools-II v2.485 was used for normalization and visualization of *JrFAD* gene expression levels, thereby revealing their expression patterns across different tissues and developmental stages [47].

### 4.9. Lipid Extraction and Untargeted UHPLC–MS/MS Analysis

Untargeted lipidomic analysis was performed using an MTBE-based extraction method followed by UHPLC–MS/MS. Briefly, 100 mg of frozen walnut kernel tissue powder was mixed with 0.75 mL methanol and 2.5 mL methyl tert-butyl ether (MTBE), incubated for 1 h at room temperature with shaking, and phase separation was induced by adding 0.625 mL MS-grade water. After centrifugation at 1000× *g* for 10 min, the upper organic phase was collected, and the lower phase was re-extracted with MTBE/methanol/water (10:3:2.5, *v*/*v*/*v*). The combined organic phases were dried and reconstituted in 100 μL isopropanol for UHPLC–MS/MS analysis. Lipid profiling was performed using a Vanquish UHPLC system coupled to an Orbitrap Q Exactive HF mass spectrometer (Thermo Fisher Scientific, Waltham, MA, USA) with a Thermo Accucore C30 column (150 × 2.1 mm, 2.6 μm). Chromatographic separation was achieved at 40 °C with a flow rate of 0.35 mL min^−1^ using acetonitrile/water (60:40, *v*/*v*) containing 10 mM ammonium acetate and 0.1% formic acid as mobile phase A and acetonitrile/isopropanol (10:90, *v*/*v*) containing the same additives as mobile phase B. The mass spectrometer was operated in negative-ion mode over an *m*/*z* range of 114–1700, with normalized collision energies of 22, 24, and 28 eV. Raw data were processed using Compound Discoverer 3.1 for peak alignment, peak picking, and relative quantification, with retention-time tolerance of 0.2 min, mass tolerance of 5 ppm, signal-intensity tolerance of 30%, signal-to-noise ratio of 3, and minimum peak intensity of 100,000. Peak intensities were normalized to total spectral intensity, molecular formulas were predicted based on precursor, adduct, and fragment-ion information, and lipid features were putatively annotated by matching against the LIPID MAPS and LipidBlast databases. Because authentic standards and isomer-specific confirmation were not available for all detected features, the resulting annotations were considered putative, and the normalized LC–MS/MS signals were interpreted as relative abundances of extractable lipid-related features rather than absolute fatty-acid concentrations or the fatty-acid composition of storage triacylglycerols.

### 4.10. WGCNA and Gene–Metabolite Correlation Analysis

To characterize gene co-expression patterns during walnut kernel development, weighted gene co-expression network analysis (WGCNA) was performed using the normalized expression profiles of 21,586 genes from 15 samples representing five developmental stages, with three biological replicates per stage. A soft-thresholding power of 18 was selected to construct the adjacency matrix and topological overlap matrix. Genes were clustered using hierarchical clustering, with the minimum module size set to 50. Module eigengenes were correlated with developmental stages using Pearson correlation analysis.

Functional enrichment analysis of the cyan module was performed using the clusterProfiler package v [4.2.0] in R v4.1.2. To further investigate the relationships between *JrFAD* expression and developmental variation in lipid-related LC–MS features, the expression profiles of the 21 *JrFAD* genes were integrated with the normalized abundances of five representative lipid-related LC–MS features, including palmitic acid, stearic acid, oleic acid, putatively annotated C18:2 fatty-acid feature, and putatively annotated C18:3 fatty-acid feature, across the same 15 samples. Pearson correlation coefficients were calculated between the expression levels of the 21 *JrFAD* genes and the normalized signal intensities of the five lipid-related LC–MS features across the 15 matched samples, resulting in 105 gene–feature tests. The corresponding *p* values were adjusted for multiple testing using the Benjamini–Hochberg procedure, and FDR < 0.05 was considered statistically significant. The complete correlation statistics are provided in Table S9. Predicted protein association analysis of JrFAD proteins was performed using STRING v12.0 with Juglans regia as the reference organism. JrFAD protein sequences were mapped to the corresponding STRING entries, and successfully mapped proteins were used for network construction.

## 5. Conclusions

In this study, a total of 21 *FAD* gene family members were systematically identified in the walnut genome and classified into five subfamilies. The results revealed that members within the same subfamily exhibited high conservation in conserved motifs, domains, and gene structures, whereas distinct functional divergence was observed among different subfamilies. The 21 *JrFAD* genes were unevenly distributed across nine chromosomes, with WGD/segmental duplication serving as the primary driver of family expansion. All duplicated gene pairs have undergone strong purifying selection, indicating that their coding sequences are evolutionarily constrained and relatively conserved throughout evolution. Furthermore, the promoter regions of these genes are enriched with numerous regulatory elements involved in light signaling, hormonal regulation, and stress responses, suggesting the potential for complex transcriptional regulation. Expression pattern analysis revealed that *JrFAD3.1* and *JrFAD2.3* were specifically and highly expressed during the rapid oil accumulation phase in kernels, implicating that they are candidate genes associated with unsaturated fatty acid biosynthesis at the transcript level. Collectively, this study provides useful information for further dissecting the biological functions of the walnut *FAD* gene family and their regulatory roles in lipid metabolism. Future research should focus on functional validation and regulatory network dissection of key *JrFAD* genes, employing approaches such as qRT-PCR, heterologous expression, and gene editing, combined with association analysis between natural genetic variation and oil quality traits in walnut populations, to provide scientific evidence and concrete strategies for the improvement of walnut oil quality and unsaturated fatty-acid composition.

## Data Availability

Data is contained within this article and the Supplementary Materials. The exact JRELIAO1 genome assembly and genome-wide structural annotation used in this study are publicly available through Figshare. The genome assembly FASTA file is available under DOI 10.6084/m9.figshare.33327072, and the complete genome annotation GFF3 file is available under DOI 10.6084/m9.figshare.33350970. The raw whole-genome sequence data of walnut, comprising Illumina short reads, HiFi reads, and Hi-C interaction reads, along with RNA-seq samples from developing seeds at five developmental stages, have been deposited in the NCBI under BioProject accession number PRJNA1274675, The raw genomic sequencing data generated for the ‘JRELIAO1’ walnut genome are publicly available in the NCBI Sequence Read Archive under BioProject PRJNA1274675 (SRA Study SRP618963), including SRR38275583 (PacBio Sequel II whole-genome sequencing), SRR38275584 (Illumina HiSeq 4000 Hi-C sequencing), and SRR38275585 (Illumina HiSeq 4000 Hi-C sequencing). SAMN51215662–SAMN51215664 for S1 stages, SAMN51215668–SAMN51215670 for S2 stages, SAMN51215677–SAMN51215679 for S3 stages, SAMN51215686–SAMN51215688 for S4 stages, and SAMN51215692–SAMN51215694 for S5 stages.

## Supplementary Materials

The following supporting information can be downloaded at https://www.mdpi.com/article/10.3390/ijms27177879/s1.
