# Supplementary material for: Genome-Wide Identification and Bioinformatics Analysis of the FAD Gene Family in Walnut (Juglans regia L.)
Source: Int J Mol Sci. 2026 Sep 3;27(17):7879. doi: 10.3390/ijms27177879 (PMC13566554; doi:10.3390/ijms27177879)
Supplement: Supplementary file 1 [file ijms-27-07879-s001.zip › Supplement Figures.pdf]

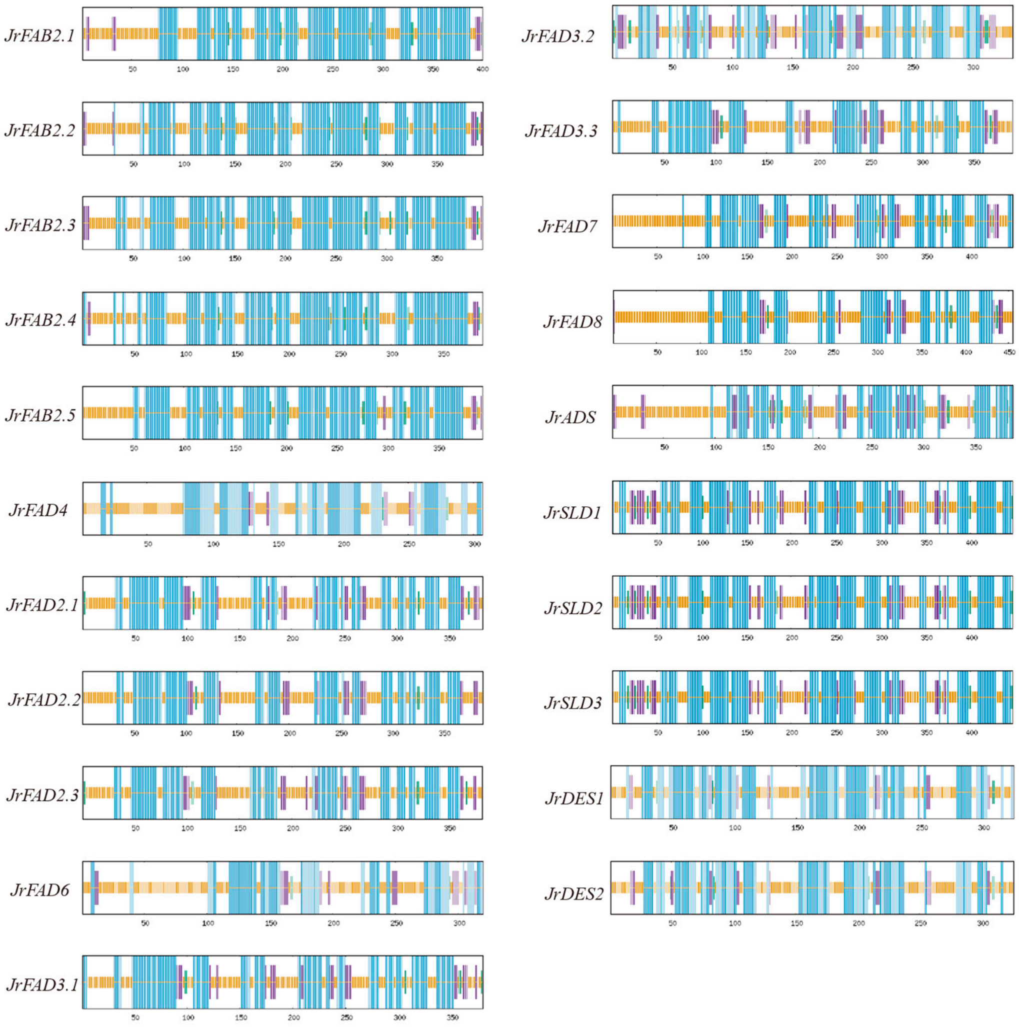

Figure S1. Prediction of secondary structure of JrFAD proteins. Note: Blue indicates the α-helix; purple indicates the extension chain; green indicates β-angle; orange indicates random curls.

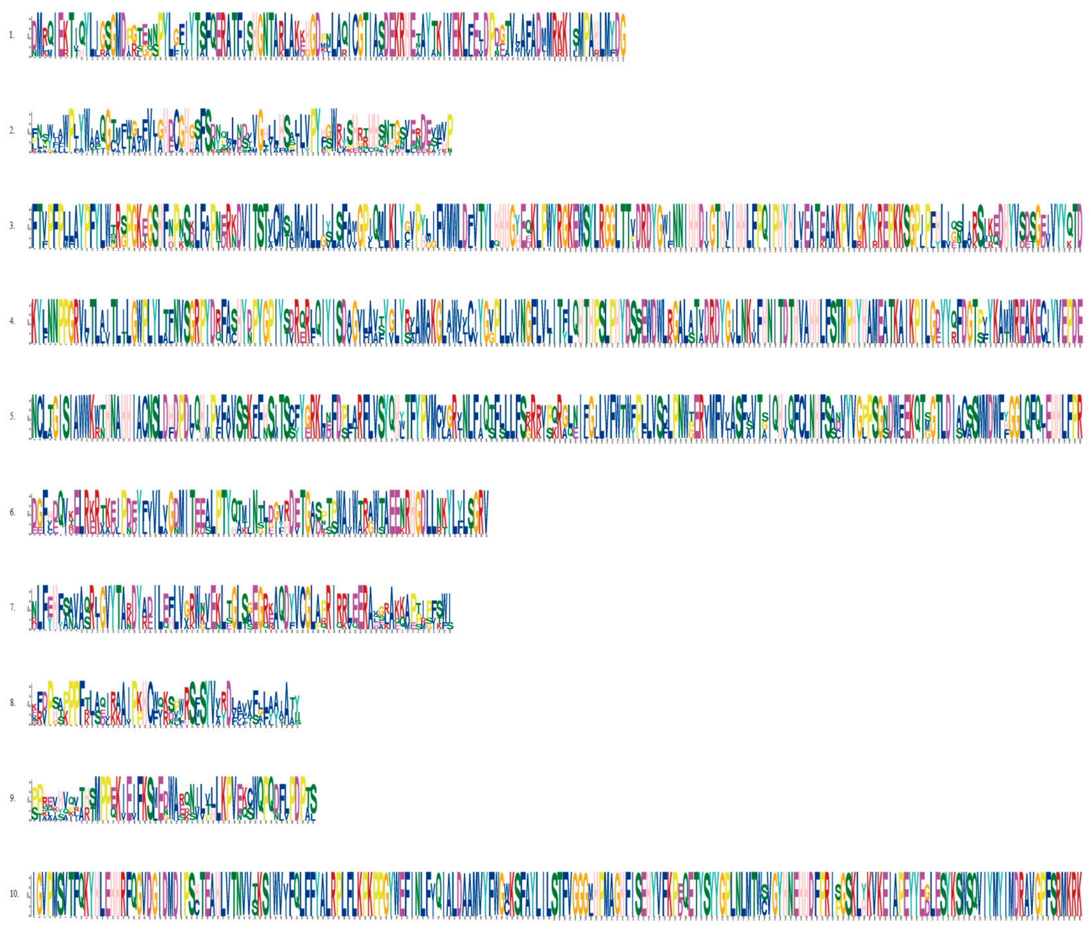

Figure S2. Sequence logos of conserved motifs in JrFAD proteins.

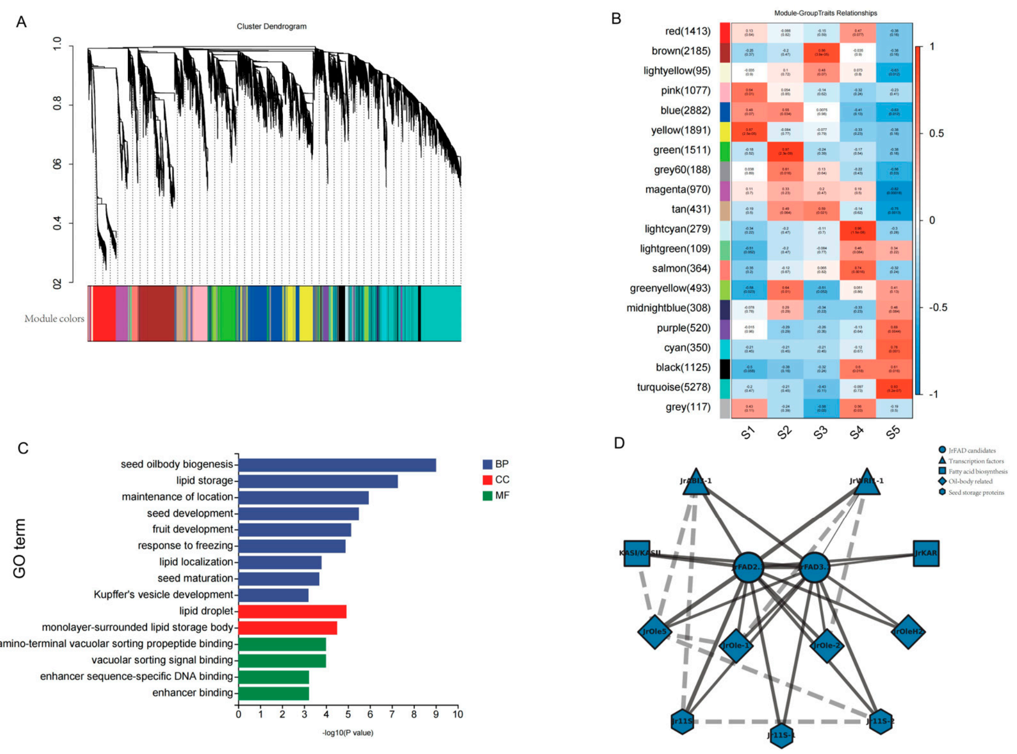

Figure S3. Weighted gene co-expression network analysis and functional characterization of the cyan module during walnut kernel development. A. Hierarchical clustering dendrogram of the expressed genes and corresponding WGCNA module assignments. B. Heatmap showing the correlations between module eigengenes and the five kernel developmental stages. C. Representative GO terms enriched among genes in the cyan module. BP, biological process; CC, cellular component; MF, molecular function. D. *JrFAD*-centered local co-expression network constructed using 13 lipid metabolism- and seed maturation-related genes from the cyan module.

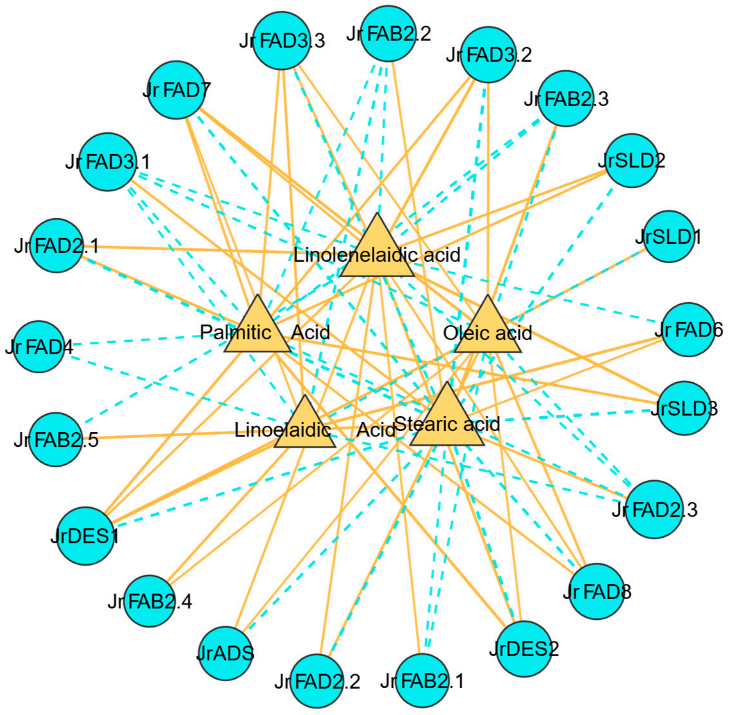

Figure S4. Correlation network between *JrFAD* gene expression and representative fatty acid metabolites during walnut kernel development.

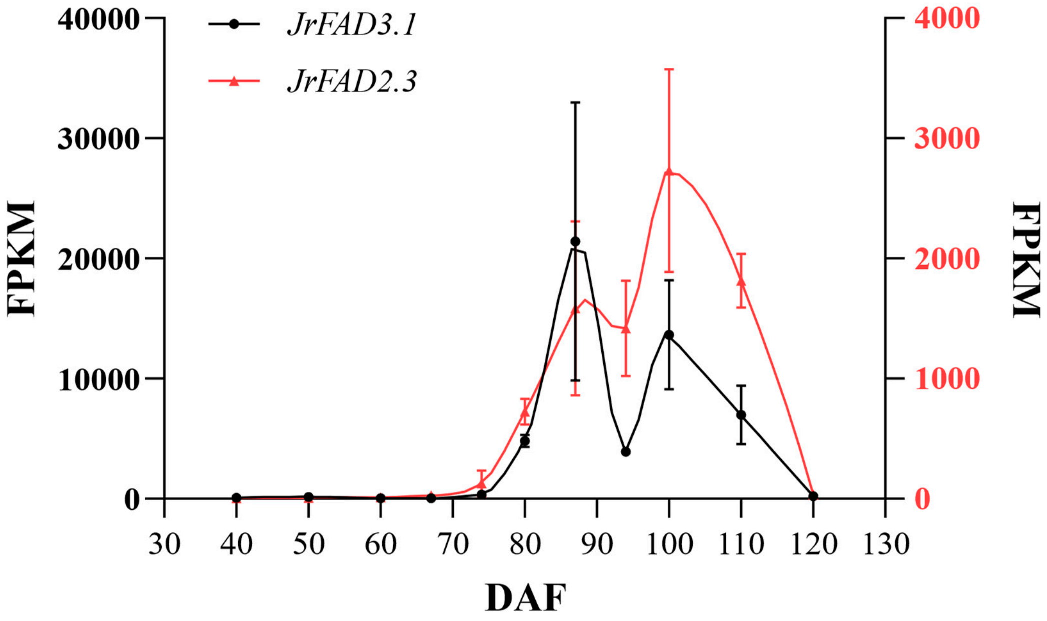

Figure S5. Expression patterns of *JrFAD3.1* and *JrFAD2.3* during walnut kernel development.

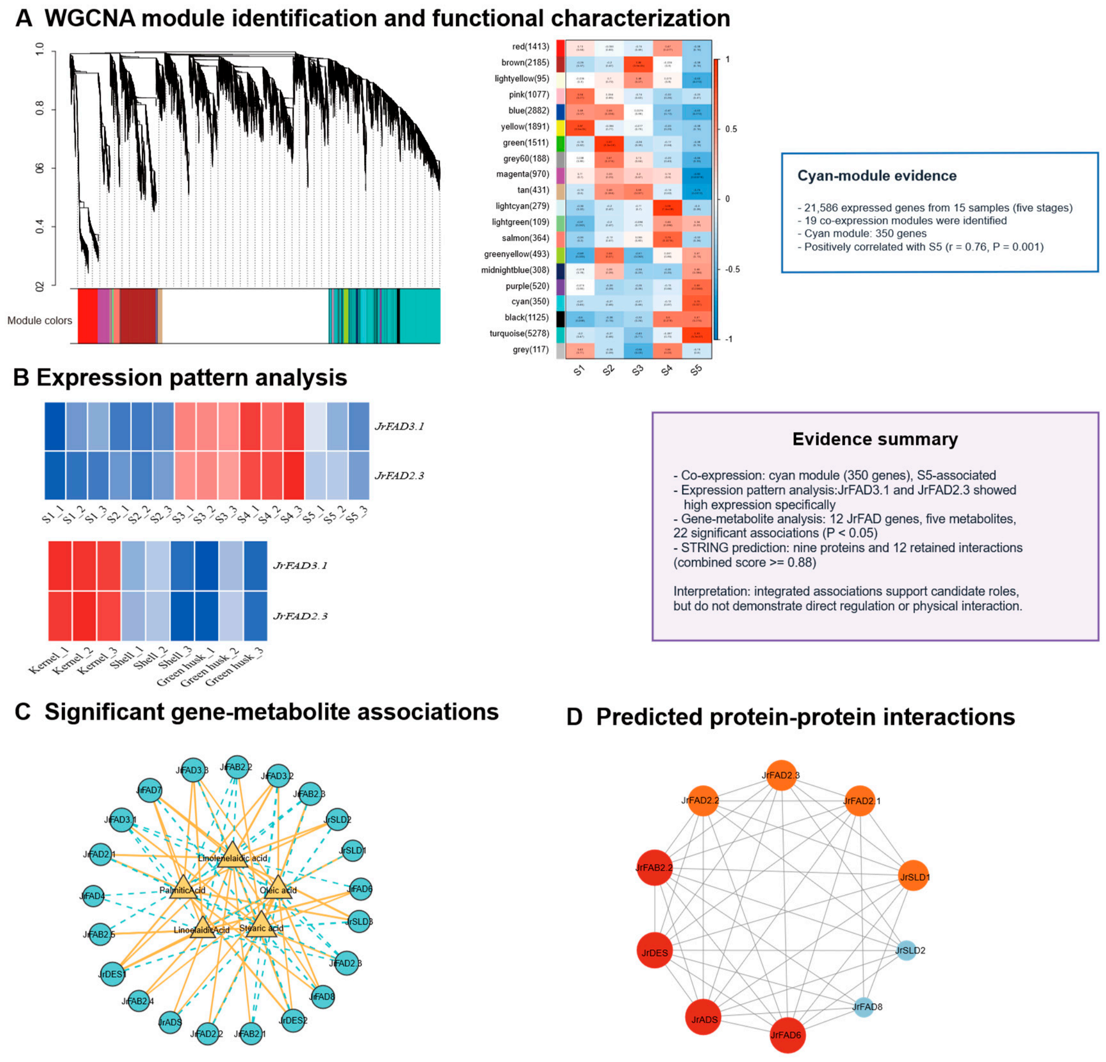

Figure S6. Integrated evidence supporting the potential roles of *JrFAD* genes during walnut kernel development. A. Weighted gene co-expression network analysis of 21,586 expressed genes from 15 kernel samples representing five developmental stages. B. Heatmap of *JrFAD3.1* and *JrFAD2.3* expression across different developmental stages of walnut kernels and in various tissues. C. Correlation network between *JrFAD* gene expression and five representative fatty acid metabolites. D. STRING-based prediction of protein–protein interactions among FAD-related proteins.
